# Normal and Abnormal Sharp Wave Ripples in the Hippocampal-Entorhinal Cortex System: Implications for Memory Consolidation, Alzheimer's Disease, and Temporal Lobe Epilepsy

**DOI:** 10.3389/fnagi.2021.683483

**Published:** 2021-06-28

**Authors:** Zhi-Hang Zhen, Mo-Ran Guo, He-Ming Li, Ou-Yang Guo, Jun-Li Zhen, Jian Fu, Guo-Jun Tan

**Affiliations:** ^1^Department of Neurology, The Second Hospital of Hebei Medical University, Shijiazhuang, China; ^2^Neurological Laboratory of Hebei Province, Shijiazhuang, China; ^3^Department of Biology, Boston University, Boston, MA, United States; ^4^Department of Emergency Surgery, The Second Hospital of Hebei Medical University, Shijiazhuang, China

**Keywords:** sharp wave ripple, hippocampal-entorhinal cortex system, Alzheimer's disease, temporal lobe epilepsy, oscillation

## Abstract

The appearance of hippocampal sharp wave ripples (SWRs) is an electrophysiological biomarker for episodic memory encoding and behavioral planning. Disturbed SWRs are considered a sign of neural network dysfunction that may provide insights into the structural connectivity changes associated with cognitive impairment in early-stage Alzheimer's disease (AD) and temporal lobe epilepsy (TLE). SWRs originating from hippocampus have been extensively studied during spatial navigation in rodents, and more recent studies have investigated SWRs in the hippocampal-entorhinal cortex (HPC-EC) system during a variety of other memory-guided behaviors. Understanding how SWR disruption impairs memory function, especially episodic memory, could aid in the development of more efficacious therapeutics for AD and TLE. In this review, we first provide an overview of the reciprocal association between AD and TLE, and then focus on the functions of HPC-EC system SWRs in episodic memory consolidation. It is posited that these waveforms reflect rapid network interactions among excitatory projection neurons and local interneurons and that these waves may contribute to synaptic plasticity underlying memory consolidation. Further, SWRs appear altered or ectopic in AD and TLE. These waveforms may thus provide clues to understanding disease pathogenesis and may even serve as biomarkers for early-stage disease progression and treatment response.

## Introduction

Alzheimer's disease (AD) is an age-related neurodegenerative disorder characterized by the accumulation of deposits containing β-amyloid protein (amyloid plaques) and neurofibrillary tangles in brain gray matter concomitant with progressive cognitive decline, which usually starts with deficits in episodic memory. There is also a strong relationship between AD and temporal lobe epilepsy (TLE), as epileptic seizures are often present in AD patients and amyloid plaques were first described in epileptic patients (Nicastro et al., 2016; Zarea et al., 2016), suggesting shared risk factors and pathomechanisms. For instance, the hyperexcitability associated with seizures may disrupt neural network connectivity, thereby increasing plaque burden (Lott et al., 2012; Vossel et al., 2017). Moreover, memory impairment is also a major sequela of temporal lobe seizures (Duan et al., 2020; Gourmaud et al., 2020). The hippocampus, a structure within the medial temporal lobe, is critical for encoding events as transferable units of experience and for the consolidation of these experiences into long-term memories (Sun et al., 2020). These processes are mediated by the formation of neuronal ensembles that fire in specific contexts, such as the location-specific hippocampal place cells observed following spatial navigation learning. It is believed that ensemble formation is mediated in part by use-dependent synaptic plasticity, such as NMDA receptor (NMDAR)-dependent long-term potentiation (LTP), initiated by oscillatory brain activity (rhythms) and regulated by a variety of neuromodulators such as brain-derived neurotrophic factor (BDNF), dopamine, and acetylcholine (ACh). These oscillations effectively enable both the formation and consolidation of memories through repeated circuit activation in the days and nights following learning events (Norimoto et al., 2018; Malerba and Bazhenov, 2019; Eichenlaub et al., 2020; Moon et al., 2020; Nguyen et al., 2020; Patel et al., 2020).

While resonance activity of these hippocampal ensembles in response to subthreshold oscillations provide a plausible network-level mechanism to accurately encode and retrieve information (Roach et al., 2018), long-term storage appears to require the slow recruitment of neocortical memory circuits through cortical plasticity (Farooq et al., 2019; Xu et al., 2019). However, the detailed cellular mechanisms for this memory transfer are unclear.

The hippocampus-entorhinal cortex (HPC-EC) is a key structure for spatial navigation in rodents as well as for various forms of associative learning in both animals and humans. During rest and certain stages of sleep, the HPC-EC electroencephalogram (EEG) displays a robust 4–7 Hz theta rhythm, and disruption of this theta rhythm impairs spatial learning and memory, while induced theta burst stimulation readily evokes synaptic plasticity within HPC-EC circuits. The first half of each theta cycle is devoted to computing current position using sensory information from the lateral entorhinal cortex (LEC) and path integration information from the medial entorhinal cortex (MEC) (Eichenbaum et al., 1999; Pastalkova et al., 2008; Norimoto et al., 2018; Rolls, 2020), although a clear dichotomy between MEC and LEC functions has yet to be established (Kerr et al., 2007). Moreover, place cells are part of a wider network of spatially modulated neurons present in hippocampal subregions CA1, CA3, and dentate gyrus (DG) (Eichenbaum et al., 1999) that become reactivated during “off-line” periods, possibly to consolidate acquired memories. In addition to place cells, grid cells and head direction cells in the MEC are also essential components of the neural navigation system (Sargolini et al., 2006; Giocomo et al., 2014). Hippocampal place cell output is required for the normal activation of grid cells in the EC (Bonnevie et al., 2013). In addition to spatial navigation, connections among various cells in the HPC and EC are required for a variety of other memory-guided behaviors (Buzsaki and Moser, 2013; Aronov et al., 2017) and various intrinsic oscillations are required for this functional coupling. For instance, multiple studies have shown that the temporal coordination through the HPC-EC system is maintained by hippocampal sharp-wave ripples (SWRs, 150–250 Hz), gamma rhythms (40–100 Hz), and theta rhythms (4–7 Hz) (Buzsaki and Wang, 2012; Moisa et al., 2016; Jiang et al., 2019). Moreover, these different frequency bands are involved in distinct stages of memory formation, especially episodic memory formation. In rodents, theta and gamma oscillations are hallmarks of active waking states when the animal is engaged in behaviors such as ambulation, exploration, rearing, or sniffing. Alternatively, SWRs occur mostly during non-REM sleep and quiescent immobile “off-line” states of the waking period such as during consummatory behaviors. Although hippocampal theta oscillations alone are capable of linking and segregating the firing of neuronal assemblies (for review, see Hanslmayr and Staudigl, 2014), we present evidence that SWRs are also essential for synaptoplastic processes, network reorganization, and signaling among brain structures involved in memory consolidation.

Hippocampal memory formation occurs in two stages (Buzsaki, 1989). First, a memory trace is encoded via weak synaptic potentiation in the CA3 network induced by theta oscillations during exploratory behavior. Next, synapses strengthened during exploration contribute to the generation of SWRs. These SWRs in turn trigger LTP at synaptic connection between CA3 and CA1 neurons (Sadowski et al., 2016). Dupret et al. (2010) reported that hippocampal neurons encode newly learned goal locations through the reorganization of ensemble firing patterns in the CA1 area but not in CA3, and that stabilization of CA1 ensembles requires NMDAR-dependent synaptic plasticity. Further, Norimoto et al. (2018) reported that SWRs can also induce long-term depression (LTD) and that disruption of SWRs suppresses spontaneous synaptic downregulation and impairs learning and memory. Collectively, these findings strongly suggest that SWRs contribute to memory function through bidirectional modulation of synaptic strength in the hippocampus.

Sharp wave ripples are among the most synchronous spontaneous population patterns in the mammalian brain, and recent evidence suggest that these waves serve to reactivate neurons encoding episodic memories such as place cells to promote stabilization (a cellular correlate of memory consolidation) and also contribute to the planning of future actions by generating ordered neuronal firing sequences (Buzsaki, 2015; Foster, 2017; Oliva et al., 2018). The population bursts underlying SWRs emerge within CA2–CA3a recurrent collaterals and spread to CA3b and CA3c (Oliva et al., 2016). It may be phase-coupled with a power spectral peak in the slow gamma band originating from the CA3, which in turn determines information flow in the HPC-EC system (Kitanishi et al., 2015). It is believed that SWR power is associated with higher fidelity replay of past experiences and of place cell trajectories (Carr et al., 2012). In line with this notion, disruption of SWRs and/or gamma oscillation in the HPC-EC of experimental animals and humans causes severe memory impairment (Le Van Quyen et al., 2010; Jadav et al., 2012; Fernandez-Ruiz et al., 2019, 2021; Hollnagel et al., 2019; Jones et al., 2019; Mendes et al., 2019).

Due to the well-established functional relationship of SWRs and/or gamma oscillations in the HPC-EC system to memory, the current review is divided into three main sections. First, we describe the neural circuits of the HPC-EC system and their functions in different stages of memory formation. Second, we discuss the contributions of SWRs and associated oscillation within the HPC-EC system to memory formation and the alterations underlying memory deficits in AD and TLE. Finally, we discuss the potential utility of coherence analysis of SWRs and associated oscillation in the HPC-EC system for early diagnosis and treatment of AD and TLE.

### Neural Circuits of the HPC-EC System and Contributions to Memory

Reactivation of memory-encoding neurons (the cellular correlate of memory replay) is crucial for strengthening synaptic connections and for transforming hippocampus-dependent memories into cortex-dependent memories for longer-term storage (Wimmer and Shohamy, 2012; Buzsaki, 2015). In general, the hippocampal subfields are differentially involved in the representation of recent and remote autobiographical memories during vivid recall (Bonnici et al., 2013). Like the HPC, the EC exhibits a variety of state-dependent network oscillations that are believed to effectively organize neuronal activity in time during memory formation (Roth et al., 2016). Finally, functional connectivity between the HPC and cortex is also essential for retrieval of distant memories and their integration into neocortical networks (Winocur and Moscovitch, 2011). Recent functional magnetic resonance imaging (fMRI) studies have demonstrated that cognitive spaces defined by continuous dimensions are represented by the human HPC-EC system (big-loop recurrence) (Tavares et al., 2015; Constantinescu et al., 2016; Koster et al., 2018). Therefore, understanding how HPC-EC system orchestrates activity can provide insight into the mechanisms of for memory formation and consolidation.

**Figure 1:**
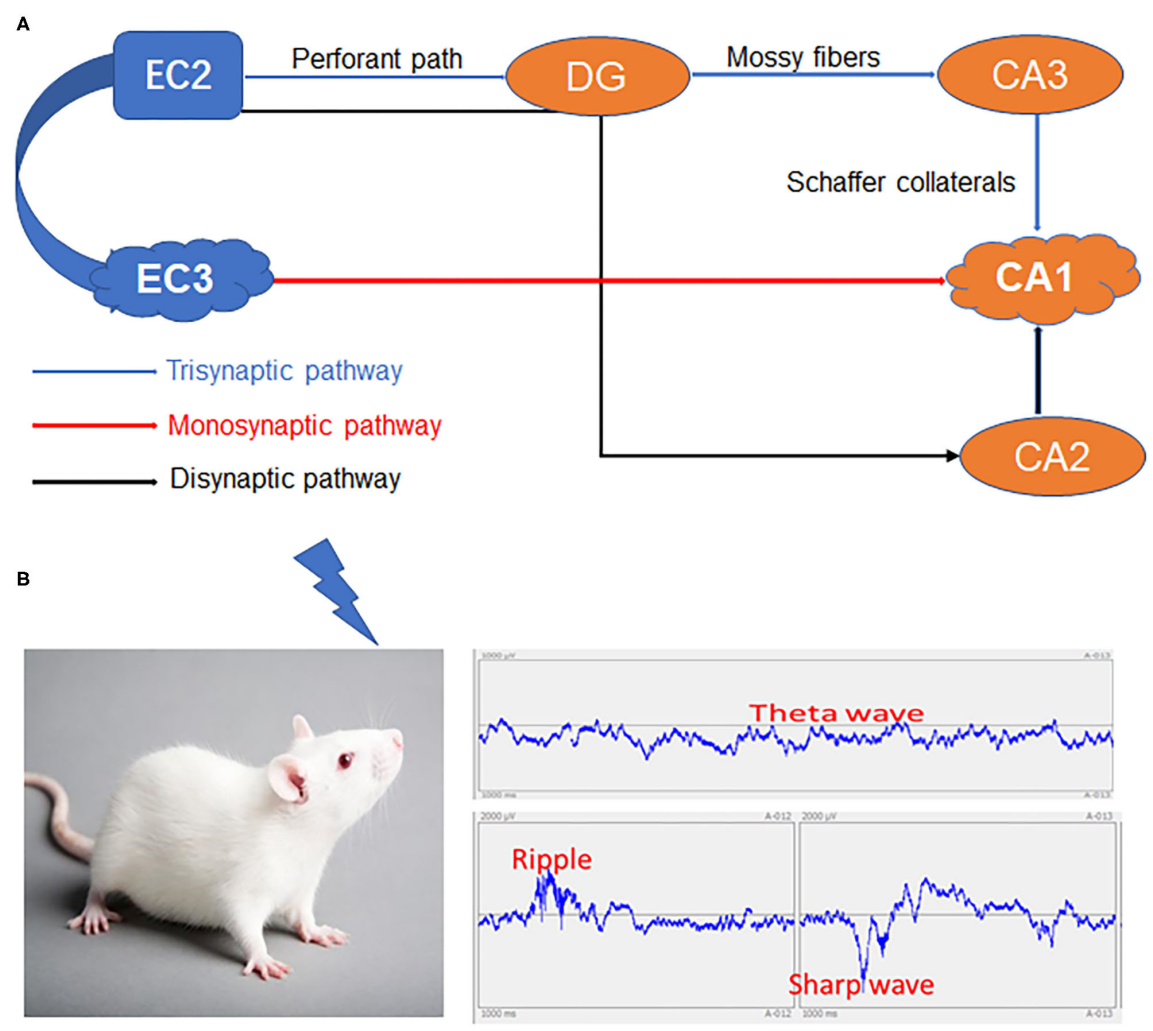
The HPC-EC system and network changes spanning from immediate memory to consolidation. **(A)** The three major circuits in the hippocampus. **(B)** Specific inputs to CA1 pyramidal cells in relation to local network oscillations. HPC, Hippocampus; DG, Dentate gyrus; EC2, Entorhinal cortex layer II; EC3, Entorhinal cortex layer III; CA1, CA3, CA2 and DG areas constitute the HPC.

Although debate persists on whether semantic information eventually becomes entirely hippocampus-independent after consolidation (Nadel and Moscovitch, 1997; Manns et al., 2003), there are several reports that even long-established episodic memories depend on the HPC-EC system (Squire, 1992; Eichenbaum and Fortin, 2005). The internal signaling flow underlying this episodic memory formation is well-described based on hippocampal circuit anatomy. The CA1 receives signals from both a monosynaptic pathway from the EC layer III (EC3) and from the EC2–DG–CA3 trisynaptic pathway. Another EC2–DG–CA2–CA1 pathway bypassing the CA3 has also been described (Kohara et al., 2014), but its involvement in memory encoding is unclear. Alternatively, numerous studies indicate that CA3 output and the synaptic strengthening between CA1–CA3 and CA1–EC3 synapses are necessary for CA1 place cell activity (Kitanishi et al., 2015) (Figure 1).

In mammals, the HPC-EC system contributes to spatial navigation by mapping relationships in situations where knowledge is physical, continuous, and consciously available (Chadwick et al., 2015). Transgenic mice with inactive EC3 inputs to the HPC demonstrated spatial working memory deficits in a trace-fear conditioning task (Suh et al., 2011), implying that this HPC–EC pathway is critical for temporal association memory, especially episodic memory as remembering event sequences is central to episodic memory (Chadwick et al., 2014). In line with these findings, recent studies also have shown that multi-voxel patterns in the EC, the human homolog of the rodent LEC, specifically reflect the temporal event structure after learning via neuronal network oscillation activity (Bellmund et al., 2019).

Although the function the HPC-EC system in episodic memory formation is only just beginning to be explored, current findings are relevant to memory dysfunction in AD and TLE. Therefore, in the following sections, we will introduce an updated model of neural oscillations in the HPC-EC system, discuss the underlying network mechanisms, and then describe possible alterations contributing to memory deficits in AD and TLE based on both *in vitro* and *in vivo* studies.

### Network Mechanisms Underlying SWRs Generation, SWRs Functions in the HPC-EC System, and SWRs Alterations in AD

Rhythmic voltage oscillations measured from the extracellular space of nervous tissue (field potentials) have long been used to assess coordinated neural activity within and among various brain regions (Einevoll et al., 2013; Norman et al., 2019). SWRs can be measured from multiple regions and correspond to distinct neural processes associated with cognition (Benthem et al., 2020; Jura et al., 2020). Hippocampal SWRs are strongly associated with memory consolidation and retrieval via interactions with the neocortex (Joo and Frank, 2018). Further, concurrent SWRs can induce synaptic potentiation between neurons *in vivo*, resulting in stronger functional coupling and SWRs coherence (King et al., 1999). In animal models, including rats and macaques, such coincident SWRs among neuronal populations are observed during quiet immobility and sleep as well as during consummatory behavior, grooming, brief interruptions in locomotion, and even during active visual exploration (O'Neill et al., 2006; Cowen et al., 2020; Hussin et al., 2020; Nokia et al., 2020; Sosa et al., 2020).

In permissive network states, extracellularly recorded SWRs are observed as large-amplitude fast oscillations in CA1 and CA3 that correspond to the synchronous depolarization of local neurons functionally coupled by synaptic plasticity (Ramirez-Villegas et al., 2018). In addition, SWRs may propagate between structures, such as the CA1 and cortex, to entrain further neuronal populations. This propagation may be essential for the cortical storage of memories, although this remains a matter of debate.

Multiple studies have shown that SWRs are generated by CA3 pyramidal cells and require initial excitation of CA1 pyramidal cells as well as the participation of parvalbumin-expressing fast-spiking inhibitory interneurons that fire independently of external inputs and propagate signals through the “output loop” into the EC (Ylinen et al., 1995; Roth et al., 2016). In addition, EC input to CA1 is crucial for local SWR bursts and long-range reactivation (replay) specifically in the quiet awake state, whereas CA3 input is essential for both (Yamamoto and Tonegawa, 2017). It is also possible that this activity is also modulated by CA2 (Oliva et al., 2016) and DG (Sasaki et al., 2018). An optogenetic study in rats found that local field potential–spike coupling between CA1 and CA3 regions during SWRs was lowest in the gamma band and that longer SWRs were preceded by increased firing in the EC (Oliva et al., 2018), incidentally the first region of the hippocampal formation affected by Aβ accumulation in AD (Harris et al., 2010).

Indeed, the same activity from CA3 that excites a large subset of CA1 pyramidal cells (Valero et al., 2017) also excites interneurons (Palop and Mucke, 2016), resulting in oscillatory excitation and inhibition of interneuron-coordinated pyramidal cell ensembles that manifest as coincident ripples. In a mouse model, soluble amyloid beta oligomers blocked the learning-induced increase in hippocampal SWRs rate and impaired spatial memory formation (Nicole et al., 2016). In addition to circuits within the HPC, SWRs have recently been observed in HPC–EC pathways (Wang and Ikemoto, 2016; Rothschild et al., 2017; Opalka et al., 2020), where they may contribute to reconsolidation of memories and hippocampal–neocortical signaling underlying memory transfer (Karimi Abadchi et al., 2020). For instance, selective elimination of early SWRs during post-training consolidation periods resulted in performance impairment in AD model animals trained on a hippocampus-dependent spatial memory task (Girardeau et al., 2009; Jones et al., 2019). In future experiments, it is essential to elucidate the mechanisms of SWR propagation and the functional significance of these signals to cortical plasticity and memory.

Fast-spiking parvalbumin-positive interneurons modulate the temporal spiking activity of pyramidal cells thought to initiate SWRs. Lower amplitude SWRs with altered temporal structure have been reported in the rTg4510 mouse model of AD, resulting in increased phase-locking of pyramidal cells and decreased phase-locking of interneurons (Witton et al., 2016). A selective decrease in excitatory synaptic drive to parvalbumin basket cells was also reported in the 5xFAD mouse model of familial AD during the early stage of amyloid pathology, a period associated with hyperactivity and SWRs disruption (Caccavano et al., 2020). These results suggest that disruption of the functional coupling between pyramidal cells and interneurons directly contributes to altered SWRs in the HPC-EC of AD model animals.

Indeed, a growing body of literature has documented altered SWRs in AD, but the exact mechanism has not yet been clarified. In an activity-dependent genetic forms, SWRs alterations may involve the abnormal transcription of the immediate early gene Npas4, which regulates a multitude of genes expressed by hippocampal CA1 pyramidal neurons that govern the integration of adult-born neurons into hippocampal circuits (Sim et al., 2013), a process essential for the maintenance of normal cognitive functioning with age. In addition, altered synaptic transmission and ionic channel modulation may contribute to aberrant plasticity and SWRs signaling in AD. The extracellular matrix is a major regulator of neuronal synaptic plasticity, and dysfunction of mature perineuronal nets can aberrantly increase SWRs frequency (Sun et al., 2018). Besides, dopaminergic activation is likely to reorganize cell assemblies during SWRs (Miyawaki et al., 2014). Further, reduced Nav1.1 level and parvalbumin cell dysfunction contribute to abnormalities in oscillatory rhythms, SWRs network synchrony, and memory in AD model animals (Verret et al., 2012).

Conversely, modulating interneuron-dependent network alterations and synaptic plasticity induced by SWRs could be a potential therapeutic strategy to improve HPC-EC system function in early-stage AD.

### Network Mechanisms Underlying SWRs and Alterations in TLE

According to fMRI studies, both TLE and AD demonstrate reduced resting-state activity and functional connectivity within the default mode network (DMN), a cortical system that is most active during conscious rest, suggesting shared regional network dysfunction (Luo et al., 2011). Deficits of episodic memory are also frequent in TLE (Helmstaedter, 2002; Holler et al., 2020) and other neurological disorders known to specifically affect temporal lobe circuits (Derner et al., 2018; Kilias et al., 2018; Kuhn et al., 2018). Moreover, loss of MEC layer III neurons in TLE alters intrinsic membrane and synaptic properties within associated circuits, resulting in hyperexcitability (Tolner et al., 2007). Typically, TLE is characterized by profound changes in hippocampal and parahippocampal network circuitry resulting in spontaneous recurrent seizures and interictal activity in both humans and animal models (Sidhu et al., 2013; Sloviter and Bumanglag, 2013; Kuhn et al., 2018).

Further, the network- and synapse-level changes required for encoding and retrieval of memory within HPC-EC circuits may be conducive to the generation of hyperactivity if compensatory control processes are disrupted. Single neurons in the human EC alter their spatial tuning to target relevant memories for retrieval (Qasim et al., 2019). Activating DG GABAergic interneurons in the HPC-EC can effectively inhibit the spread of ictal seizures and largely rescue behavioral deficits in TLE mice (Lu et al., 2016). These findings suggest that facilitated transfer of information between the HPC and EC may promote the construction of epileptogenic circuits. Collectively, such studies implicate altered network oscillations in TLE and associated memory deficits.

Both synchronization and desynchronization occur during memory encoding, with synchronization mostly described within the medial temporal lobe and desynchronization in the neocortex (Hanslmayr and Staudigl, 2014; Parish et al., 2018). Intriguingly, functional connectivity between the EC and HPC has also been associated with the temporal coupling of brain oscillations, such as theta and gamma waves, which selectively modifies both feed-forward connections (mossy fibers and synapses of the perforant path from DG to CA3) and feedback connections (CA3 recurrent collaterals) via different mechanisms (Chauviere, 2019). Theta is already impaired early after status epilepticus (SE), and circuit remodeling post-SE may decrease the cooperation between theta generators. Impairment of parvalbumin basket cells in TLE rats resulted in firing patterns poorly coordinated with the theta cycle as well as reduced gamma oscillations, probably related to lower input strength from the CA3–CA1 Schaffer collaterals and altered theta oscillations (Lopez-Pigozzi et al., 2016). Several related reviews have also emphasized the role of theta–gamma coupling as a crucial neural code for mnemonic processes within the HPC-EC network (Colgin, 2015; Heusser et al., 2016). Hence, here we introduce a potential role for SWRs within the HPC-EC network in TLE.

Sharp wave ripples in rat HPC-EC are of particular interest in epilepsy research for two reasons. First, patients with TLE have memory impairments and these waveforms are strongly involved in memory consolidation. Second, the epileptic brain generates pathological high-frequency oscillations (HFOs) resembling SWRs (Foffani et al., 2007). However, isolated SWRs associated with memory consolidation are markedly reduced in TLE model rats (Marchionni et al., 2019). Nonetheless, it is difficult to effectively distinguish between pathological HFOs and normal SWRs. Bragin et al. described two similar types of HFOs recorded from the HPC-EC of patients with mesial TLE. A key distinction between pathological HFOs and normal ripples is that the former are readily recorded from DG, whereas SWRs are not observed in the DG under normal conditions (Bragin et al., 1999a,b; Flynn et al., 2015). Therefore, pathological HFOs typically occur in association with interictal EEG spikes, are associated with regions capable of generating spontaneous seizures, and appear more disorganized temporally and spatially compared to memory- associated SWRs (Staba et al., 2002; Foffani et al., 2007; Jacobs et al., 2008).

To understand how SWR-like events may contribute to TLE, Ewell (2018) compared hippocampal single-cell activity during physiological and pathological SWRs measured from single cell recordings in normal and epileptic rats with different memory abilities and found that SWRs in epileptic rats had greater spectral power in high-frequency bands and that cell-specific synaptic inputs govern the firing selectivity of CA1 pyramidal cells during SWRs. Liotta et al. investigated the relation between recurrent epileptiform discharges (REDs) and SWRs, and found that the cholinergic agonist nicotine, which is known to facilitate LTP induction, dose-dependently transformed SWRs into REDs in the hippocampus. This transition was associated with reduced inhibitory conductance in CA3 pyramidal cells, suggesting that recruitment of inhibitory cells during SWRs may prevent hyperexcitation and seizure generation (Liotta et al., 2011). Similarly, a series of epileptic animal model and brain slice studies reported a feed-forward propagation pathway of ictal discharges from DG to MEC circuits, which may also provide clues to therapeutic measures for TLE involving HPC–EC pathways (Carter et al., 2011; Lu et al., 2016). How then do these aberrant SWRs disrupt episodic memory in TLE?

While brain network structure obviously influences neural activity patterns, it is also true that these activity patterns can modify network organization through structural and synaptic plasticity. This reciprocal interaction makes it especially difficult to distinguish whether abnormal SWR activity contributes to TLE pathogenesis or is merely a consequence of TLE-associated network dysfunction (Warren et al., 2017). Dysfunctional synaptic plasticity may drive brain network disruption in TLE, but the underlying mechanisms are unclear. One possibility is disruption of the normal excitatory/inhibitory synaptic balance. For instance, impaired GABAergic transmission could induce hyperexcitability and epileptiform activity, and also disrupted normal hippocampal LTP induction (Lei et al., 2016). Similarly, deficient AMPA receptor palmitoylation, a major regulator of surface expression and phosphorylation state, resulted in hyperexcitability and seizures (Itoh et al., 2018). Hence, a decrease in GABAergic inhibitory activity and/or increased glutamatergic excitatory activity could promote neuronal hyperexcitability and epileptogenesis, as well as impair hippocampal LTP, contributing to learning and memory deficits. In fact, augmenting GABAergic activity can effectively preserve network stability in the epileptic brain (Itoh et al., 2018). Further studies on the contributions of SWRs to TLE and the underlying mechanisms may provide clues to novel antiepileptic drugs. Interestingly, SWRs appear to contribute to the formation of memory engrams and erasure of irrelevant information during slow wave sleep by increasing the net synaptic depression necessary to increase neuronal responsiveness (Norimoto et al., 2018). Notably, optimal network architecture, characterized by efficient information processing and resilience, and reorganization after damage strictly depend on the balance between these forms of plasticity (Stampanoni Bassi et al., 2019), and patterns of neuronal activity including SWRs may differentially shape synaptic connections.

Nevertheless, because SWRs/HFO display substantial frequency overlap with static and progressive epileptogenic disturbances, the practical utilization of these waveforms for presurgical evaluation of epileptic foci has not yet been achieved (Engel et al., 2009; Frauscher et al., 2017). As SWRs are usually associated with robust network inhibition, they may represent an intrinsic, dynamic mechanism to modulate the threshold for seizure induction (Liotta et al., 2011). In either case, we believe that HFOs in the HPC-EC EEG could be used as a non-invasive marker to predict the development of epilepsy following potentially epileptogenic cerebral insults or prolonged seizures, and thus, facilitate more timely initiation of preventive therapy.

Taken together, these results help us better understand the role of excitatory and inhibitory neuronal circuits in the generation of SWRs and to define the subtle border between physiological and pathological synchrony in the TLE brain.

## Conclusion

**Figure 2:**
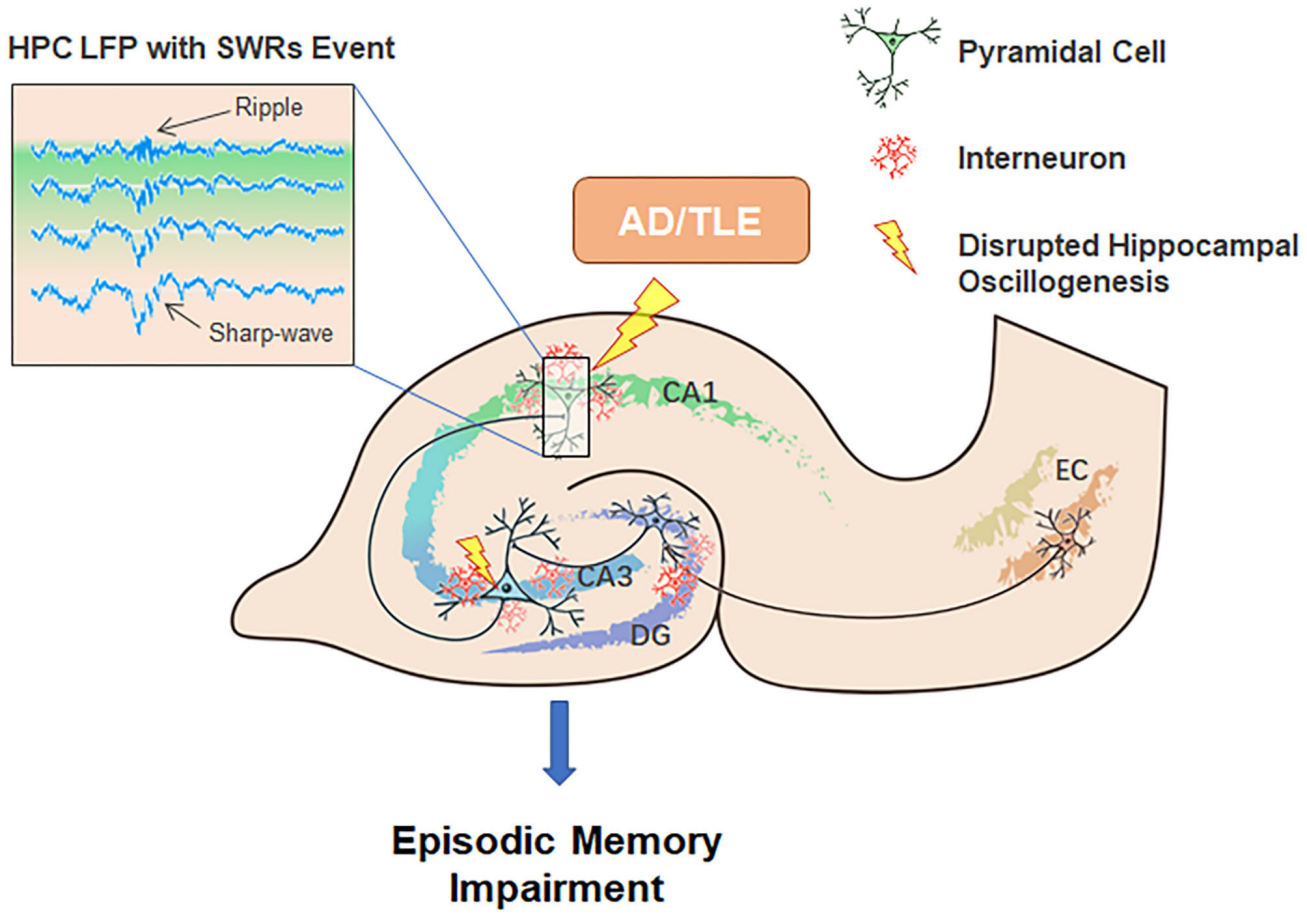
Potential contributions of neuronal network oscillations within the HPC-EC system to episodic memory encoding, consolidation, and the pathogenesis of AD and TLE. Neuronal network oscillations, especially SWRs, are disturbed in AD and TLE.

The HPC-EC system is likely critical for a myriad of diverse behavioral tasks in addition to supporting cognitive processes underlying spatial navigation. After hippocampal neurons respond to the acquisition of novel information, SWRs will trigger transient network synchronization by modulating synaptic plasticity. Our current review suggests dual roles for SWRs in memory consolidation. On one hand, SWRs facilitate LTP amongst active cells that encode spatial or episodic information, while on the other, SWRs also trigger LTD at certain synapses from the HPC-EC system to neocortex, which may contribute to memory refinement, especially of episodic memories (Yamamoto and Tonegawa, 2017). We suggest that an imbalance between these processes may underlie AD and epileptogenesis (Figure 2).

Therefore, SWRs may coordinate brain-wide networks involved in system consolidation, including hippocampus and other associated brain regions such as perirhinal cortex (Doron et al., 2020). Future studies are warranted to examine the neurophysiological factors that indirectly affect the balance between excitatory and inhibitory processes for memory induction and consolidation, such as somatostatin neurons (Sharma et al., 2020), since an abnormal sleep-wake cycle drives AD neurodegeneration and sleep deprivation increases of the spread of AD pathology (Holth et al., 2019). To what extent SWRs function differently among neuronal subtypes, and whether such functional heterogeneity is directly related to the expression of activity-dependent immediately early genes such as Npas4, ARNT2, and Homer1a are also important issues for future research. Finally, much additional research is needed to elucidate how an episodic memory is encoded by neuronal ensembles.

In summary, patients with AD and epilepsy, especially TLE, can show accelerated cognitive decline and may benefit from antiepileptic treatments that target network hyperexcitability due to aberrant SWRs. How SWRs and related oscillations reorganize and consolidate circuits under non-pathological conditions may provide clues to the contributions of these signals to AD, TLE, and associated cognitive deficits. Further studies combining neurophysiological and fMRI measures are required to better understand the spatial and temporal relationships among brain oscillations and plastic changes at circuit and network levels in health and disease.

## Author Contributions

Z-HZ and M-RG wrote the original draft. H-ML and O-YG modified the figures. J-LZ, JF, and G-JT revised the manuscript. All authors contributed to the article and approved the submitted version.

## Conflict of Interest

The authors declare that the research was conducted in the absence of any commercial or financial relationships that could be construed as a potential conflict of interest.

## Abbreviations

SWRs: Sharp wave ripples
AD: Alzheimer's disease
HPC-EC: hippocampal-entorhinal cortex
DG: dentate gyrus
TLE: temporal lobe epilepsy
NMDAR: NMDA receptor
LTP: long-term potentiation
BDNF: brain-derived neurotrophic factor
Ach: acetylcholine
EEG: electroencephalogram
LEC: lateral entorhinal cortex
MEC: medial entorhinal cortex
EC3: entorhinal cortex layer III
fMRI: functional magnetic resonance imaging
HFOs: high-frequency oscillations.
